# To Dr. Bradley

**Published:** 1804-07-01

**Authors:** H. L. Helsham

**Affiliations:** Stoke Ferry, Norfolk


### To Dr. BRADLEY.

Sir,

As from the symptoms of the following case, there was great reason to impute them to Hydrocephalus Internus as the cause, and as the treatment has been successful, I request the favour of your inserting it in the Medical and Physical Journal.

I am, &c.

H. L. HELSHAM, M.D.

*Stoke Ferry, Norfolk, May* 28, 1804.

---

July 7, 1803, I was applied to for Miss I. S. aged 13 years, who was just come from boarding school for the holidays.

There were evident marks of a slight derangement of intellect in her conversation. She complained of violent pain and throbbing in her head, and the pupil of the right eye was a deal dilated. Her pulse was full and rather slow, but there were no signs of active inflammation.

I ordered two or three leeches to be applied to the temples, her head to be shaved, and a blister laid over it. A pill with gr. j. of calomel to be taken every night, and a julep with tinct. castorei and spir. ammon. com. two or three times in the day. This method was regularly pursued till the 31st. the blisters renewed and kept running, and the leeches occasionally applied, without her having received any permanent benefit.

She then began to rub into the thighs and arms alternately, ʒj. of ung. hydrarg. fort. till her mouth became sore and she spat plentifully.

It was now the symptoms began to give way, and by the 21st of August she seemed to have no complaint but debility, for which she took a decoction of cinchona, and continued it unremittedly till the end of September.

Since then she has had some slight returns of head-ach, which have always been relieved by applying one or two leeches.

